# Behavioral and Synaptic Phenotypes of Female *Prdx6*^−/−^ Mice

**DOI:** 10.3390/antiox11061201

**Published:** 2022-06-19

**Authors:** Tanita Pairojana, Sarayut Phasuk, Pavithra Suresh, Ingrid Y. Liu

**Affiliations:** 1 Institute of Medical Sciences, Tzu Chi University, Hualien 97004, Taiwan; 103353117@gms.tcu.edu.tw (T.P.); 103353118@gms.tcu.edu.tw (S.P.); 104727115@gms.tcu.edu.tw (P.S.); 2 Center for Biosystems Dynamics Research, RIKEN, Kobe 650-0047, Hyogo, Japan

**Keywords:** peroxiredoxin 6, antioxidation, spatial memory, anxiety, long-term potentiation

## Abstract

Peroxiredoxin 6 (PRDX6) is expressed throughout the brain, including the hippocampus, where it plays a potential role in synaptic regulation and forming emotional and spatial memories. PRDX6 is predominantly detected in the female mouse’s hippocampus; thus, we investigate the effect of the *Prdx6* gene on behavioral phenotypes and synaptic functions using female *Prdx6* knockout (*Prdx6^−/−^*) mice. Our results demonstrate that female *Prdx6^−/−^* mice exhibited anxiety-like behavior, enhanced contextual fear memory, and impaired spatial memory. We also found increased, paired–pulse facilitation ratios, and decreased long-term potentiation (LTP) in the hippocampal region of these female *Prdx6^−/−^* mice. The present study helps to understand better the PRDX6’s role in emotional response and spatial memory formation in female mice.

## 1. Introduction

Peroxiredoxin 6 (PRDX6), a multifunctional enzyme, belongs to the PRDX family, with a primary role in cellular signaling and oxidative defense mechanisms [1,2]. PRDX6 is a unique member among all peroxiredoxins (PRDXs), distinguished by its multiple functions [3]. Functions of PRDX6 include (1) its catalytic activity, which was first discovered in the bovine ciliary body as non-selenium glutathione peroxidase [4,5] and required only a single conserved cysteine residue to reduce H_2_O_2_ [1]; (2) its ability to bind phospholipid together with its phospholipid hydroperoxide glutathione peroxidase (PHGPx) to reduce phospholipid hydroperoxide [1]; (3) its acidic-calcium independent phospholipase A2 (aiPLA2) activity to hydrolyze the *sn*-2 fatty acyl bond of phospholipids [6]; and (4) its lysophosphatidylcholine acyltransferase (LPCAT) activity to transfer a fatty acyl CoA into the *sn*-2 position of lysophosphatidylcholine (LPC) [1,6]. PRDX6 is distributed throughout the body, including several brain subregions [7]. Our recent studies reveal that male *Prdx6^−/−^* mice demonstrate spatial memory deficit and increased stress susceptibility [8,9]. Its functions and distribution in the brain suggest that PRDX6 could be a key molecule in regulating stress-coping mechanisms and cognitive functions. However, whether these functions of PRDX6 are affected by gender is still unknown.

Buonora and colleagues demonstrated that PRDX6 functions in a sex-dependent manner [10]. They found the basal-level plasma PRDX6 collected from healthy subjects is higher in females than males. In contrast, plasma PRDX6 was higher in male subjects than in females suffering from traumatic brain injury (TBI) [10]. It has been reported that gender factors are correlated with differences in the synaptic plasticity and anatomical structure of the brain [11,12]. Females and males are different in the density of spines and synapses and the expression levels of specific proteins within subregions of the brain, which can affect cognitive functions and behaviors [13].

According to the above mentioned previous findings, it is not surprising that the onset and severity of brain diseases differ between males and females [14,15]. Therefore, more information regarding the impact of gender factors could help us understand the sex differences in the incidence of various brain disorders and achieve a better outcome for pharmaceutical treatment. Recently, we confirmed that PRDX6 is expressed throughout the brain, including the hippocampus, amygdala, and medial prefrontal cortex [16]. In addition, it appears to regulate emotional response and spatial memory formation in male mice [8]. Therefore, whether the *Prdx6* gene influences stress-coping mechanisms [17,18] and memory performance [19] in females is intriguing. Thus, in the present study, we investigated the function of PRDX6 in the synaptic and behavioral phenotypes of female mice.

## 2. Materials and Methods

### 2.1. Animals

A total of 60 mice, 12–14 weeks old, with 29 female wild-type controls and 31 female knockouts, in 3 batches, were used in this study. We obtained the *Prdx6* (Gene ID: 11758) knockout (*Prdx6^−/−^*) mice from Dr. Shun-Ping Huang’s lab at Tzu Chi University, originally purchased from the Jackson Laboratory (#005974 B6.129-*Prdx6* *^tm1Pgn^*/pgn, Bar Harbor, ME, USA). The *Prdx6* Mutant mice were backcrossed with C57BL/6J mice for more than ten generations. We bred all mice by mating a *Prdx6^+^^/−^* male with two *Prdx6^+^^/−^* females or intercrossing mice harboring the same genotypes. We confirmed the genotypes of mice by using real-time polymerase chain reaction (RT-PCR) with a specific primer (Primer sequence; #366 (reverse): 5′-CTTTGAACAGAACCAGGCAGG-3′, #368 (forward): 5′-CAGGATGGAGCCTCTATGCC-3′ and #369 (forward): 5′-TGGCTTCTGAGACGGAAAGAA-3′). All mice were maintained in the laboratory animal center of Tzu Chi University. Mice were weaned at 3–4 weeks old and housed as groups, each with 2–5 same-sex littermates in plastic cages with corncob bedding at a constant room temperature of 22 ± 2 °C, with 12 h light/dark cycle and ad libitum access to food and water. All experimental procedures were reviewed and approved by the Institutional Animal Care and Use Committee of Tzu Chi University, Taiwan (approval #104099-A, 24 January 2018), and followed the Taiwan Ministry of Science and Technology (MOST) guidelines for the ethical treatment of animals.

### 2.2. Behavioral Assessments

**Figure 1:**
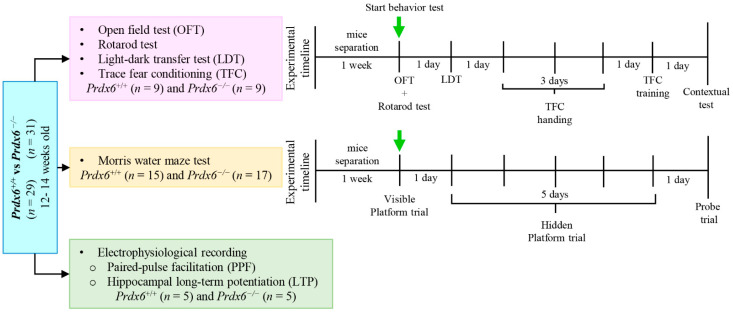
Experimental design and timeline. A total of 60 (12–14 week-old) mice, 29 *Prdx6^+/+^* and 31 *Prdx6*^−/−^, were divided into 3 batches and used in three sets of experiments. The first batch of mice was used to test a series of behavior tests, including OFT, rotarod test, LDT, and TFC. The second batch of mice was used to measure the spatial memory to MWM test. The third batch of mice was used to investigate the hippocampal synaptic plasticity by PPF and LTP recording.

Female mice were separated into individual cages one week before starting behavioral tests for habituation. We performed all behavioral experiments with dim light during the light phase in a quiet room. Experimenters were blinded to the mice groups. The first batch of mice was tested with a series of behavioral tests, including the open field test (OFT), rotarod test, light-dark transfer test (LDT), and trace fear conditioning test (TFC). All mice performed the series of behavioral tests in the same sequence. Experimental tools and equipment were cleaned with 70% ethyl alcohol between trials. We used different batches of mice in the Morris water maze (MWM) test and extracellular recording from the hippocampal slices. The experimental design is illustrated in Figure 1.

#### 2.2.1. Open Field Test (OFT)

**Figure 2:**
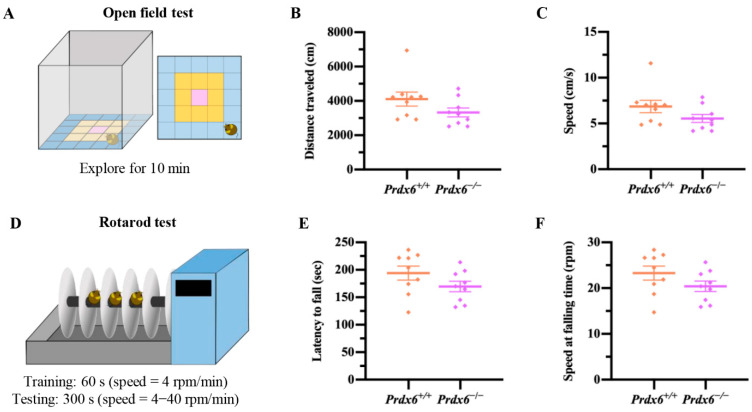
Female *Prdx6*^−/−^ mice have normal locomotor functions. (**A**) Schematic diagram of the open field arena. (**B**) The distance traveled and (**C**) moving speed in an open field arena (*Prdx6^+/+^*, *n* = 9 and *Prdx6^−/−^*, *n* = 9). (**D**) Schematic diagram of the rotarod test protocol. (**E**) Time remaining on an acceleration rotarod (s) before falling. (**F**) Rotational velocity (rpm) at the time of falling. All data are presented as mean ± SEM. PRDX6, peroxiredoxin 6.

To assess locomotion activity of the mice, an open field chamber (50 × 50 × 50 cm^3^) was placed in a quiet room with dim light and equipped with a top-down video. Mice were first located at the corner and faced the wall of the nontransparent chamber. Then mice could freely explore the chamber for 10 min. The traveling distance and speed were recorded and analyzed by video tracking software (EthoVision XT 15, Noldus Information Technology, Wageningen, The Netherlands). For anxiety-like behavior, the chamber arena was divided into three zones (outer, middle, and inner), and then tracking software was used to obtain the time spent in each zone within the 10 min exploration period. The OFT procedure is shown in Figure 2A.

#### 2.2.2. Rotarod Test

We used a rotarod test to evaluate the motor coordination with a rotarod apparatus (Ugo Basile Mouse Rotarod, #Cat. No. 47650, Ugo Basile SRL, Gemonio, Italy). First, mice were placed on the rotating rod, facing away from the direction of rotation for 60 s per trial with a constant speed of 4 rpm/min. Next, trained mice were set on the rotating rod with a start speed of 4 rpm/min for 10 s, then increased the speed to 40 rpm/min within 300 s. The latency to fall and rotated speed at the falling point were recorded and calculated. The rotarod procedure is shown in Figure 2D.

#### 2.2.3. Light-Dark Transfer Test (LDT)

**Figure 3:**
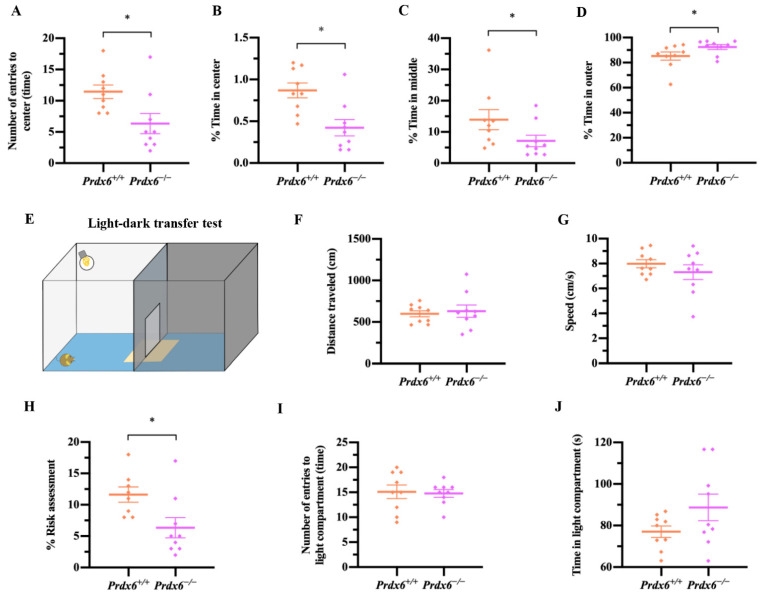
Female *Prdx6^−/−^* mice exhibit high levels of anxiety-like behavior. (**A**) Number of entries into the center of an open field arena (*Prdx6^+/+^*, *n* = 9 and *Prdx6^−/−^*, *n* = 9). Percentage of time spent in (**B**) center, (**C**) middle, and (**D**) outer areas in the open field arena. (**E**) Schematic diagram of light/dark transfer test. (**F**) The traveling distance and (**G**) moving speed in the light compartment. (**H**) Percentage of risk assessment during 10 min of exploration period. (**I**) The number of entries into the light compartment. (**J**) Time spent in the light compartment. All data are presented as mean ± SEM, * *p* < 0.05. PRDX6, peroxiredoxin 6.

Light-dark transfer test was used to measure the anxiety-like behavior. The light-dark apparatus was divided into two compartments, the dark chamber (25 (W) × 25 (L) × 35 (H) cm^3^) and the light chamber (25 (W) × 25 (L) × 35 (H) cm^3^, 700 Lux), which are connected with a sliding door located on the floor at the center of the partition. The mice were placed in a light compartment and allowed to explore the two compartments for 5 min freely. We used a camera connected with automated software (EthoVision XT 15, Noldus Information Technology, Wageningen, The Netherlands) to record moving behavior and calculate traveling speed. The total time spent in the light compartment, the risk area (3 cm length-wise × 6 cm width-wise surrounding the sliding door), and the arena outside the risk zone was recorded, and the percentage of risk assessment was calculated as: (time spent in risk area/ time spent outside risk area) × 100. The LDT procedure is illustrated in Figure 3E.

#### 2.2.4. Trace Fear Conditioning (TFC)

**Figure 4:**
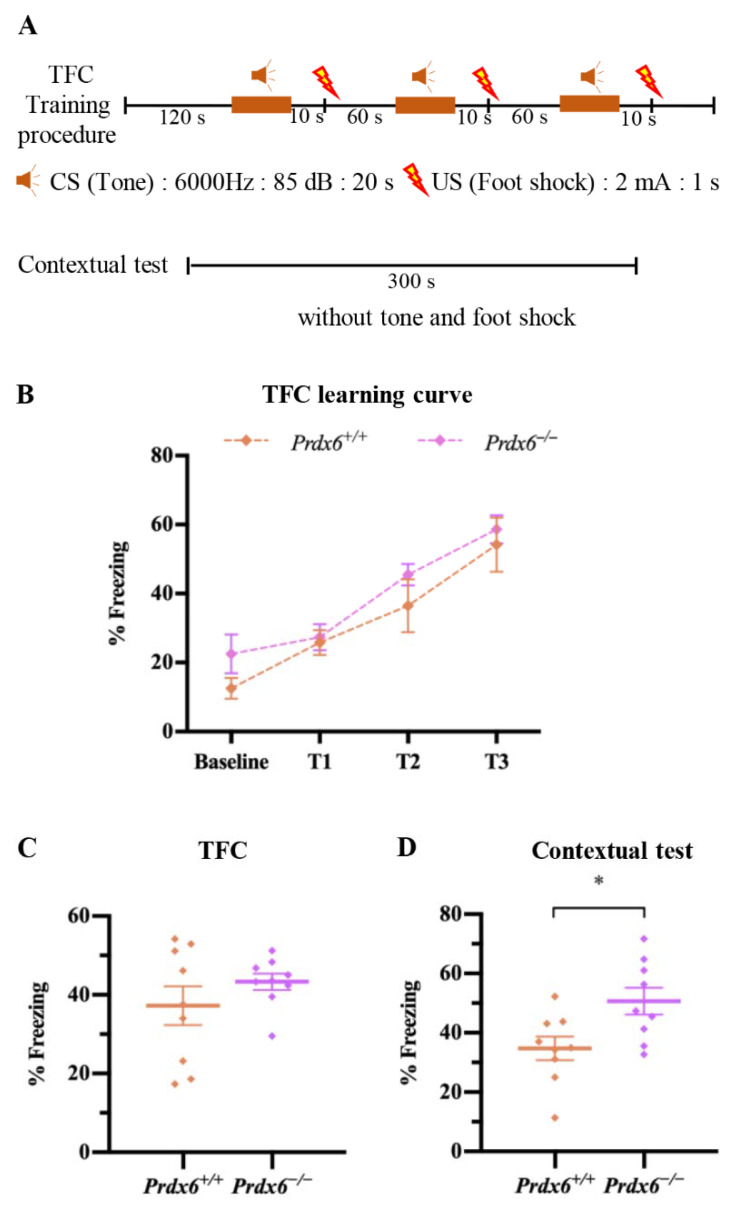
Female *Prdx6^−/−^* mice exhibited enhanced fear memory retrieval to context. (**A**) Illustration of trace fear conditioning procedure and contextual test (**B**) The learning curve for baseline and three trials of TFC training (*Prdx6^+/+^*, *n* = 9 and *Prdx6^−/−^*, *n* = 9). (**C**) The total percentage of freezing of training sessions. (**D**) The total percentage of freezing of contextual test. All data are presented as mean ± SEM, * *p* < 0.05. TFC, trace fear conditioning; PRDX6, peroxiredoxin 6.

A fear conditioning chamber (17 cm (W) × 17 cm (L) × 25 (H) cm^3^) was kept in a soundproof box with constant daylight (30 Lux). All mice were handled in this conditioning chamber for 15 min every day for 3 days. On the training day, mice were placed into the chamber for 2 min for habituation and given three pairs of conditioned and unconditioned stimuli (CS-US) (CS: 20 s, 6000 Hz, 85 dB tone and US: 1 s, 2 mA foot shock), with a 1 min inter-trial interval (ITI) between the CS-US. The total training time was 9 min. Twenty-four hours after training, mice were reexposed to the same conditioning chamber without receiving any tone or foot shock for 5 min. Figure 4A illustrates the experimental procedure of TFC. A top-view video camera was used to record the freezing behavior of the mice during training and testing sessions. The percentage of freezing behavior was analyzed by a video tracking software (EthoVision XT 15, Noldus Information Technology, Wageningen, The Netherlands). Freezing behavior was defined as no movement except respiration. The freezing time was converted to freezing percentage using the following formula: % Freezing = (total freezing time/total test time) × 100.

#### 2.2.5. Morris Water Maze Test (MWM)

**Figure 5:**
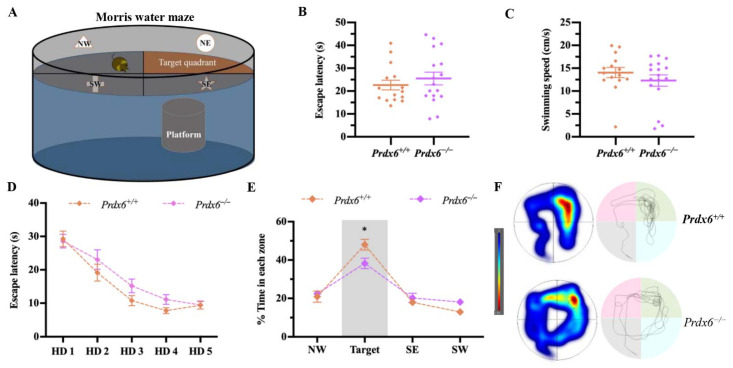
The female *Prdx6^−/−^* mice showed impaired spatial memory in the Morris water maze (MWM) task. (**A**) Illustration of a MWM. (**B**) Escape latency (time to reach the platform) and (**C**) swimming speed during the visible platform trial (*Prdx6^+/+^*, *n* = 15 and *Prdx6^−/−^*, *n* = 17). (**D**) The trial plots of escape latency for 5 days of hidden trials. (**E**) Percentage of time spent in each quadrant during a probe test. (**F**) The heat map (left panel) and swimming pattern (right panel) during a probe test. All data are presented as mean ± SEM, * *p* < 0.05, mixed-design repeated measure ANOVA followed by *Bonferroni*’s post hoc test with unpaired Student’s *t* test for individual differences between groups within each quadrant. NW, northwest; NE, northeast; SW, southwest; SE, southeast; PRDX6, peroxiredoxin 6; HD, hidden platform day.

The procedure for the MWM test was conducted according to that used in our previous publication [8]. A circular pool (diameter 110 cm) was filled with water at room temperature (21 °C ± 1 °C). The room temperature water (21 °C ± 1 °C) was mixed with a non-toxic white paint (Cat. #187203, Palmer Paint Products Inc., Troy, MI, USA) and became opaque. The pool was divided into four quadrants equally. A round platform (diameter: 10 cm, height: 21 cm) was placed in the northeast quadrant. The platform for "visible platform test" was made 1 cm above the water surface. All mice were randomly dropped to each quardrant and trained to find the visible platform within 60 s every trial and received 6 trials every day. In the hidden platform test, the platform was kept 1 cm beneath the water surface. The mice were given 60 s to find the hidden platform in each trial, and received 6 trials every day for 5 consecutive days. If the mice did not find the platform, they were guided to the platform and left there for 10 s so the mice could locate the platform by associating it with visual cues. A probe test was performed 24 h after the last “hidden platform test” trial by removing the platform from the pool. Mice were allowed to swim freely for 60 s and their swimming traces were recorded. The MWM protocol is illustrated in Figure 1 and Figure 5A. The escape latency, swimming speed, frequency reaching the platform, and percentage of time spent in each quadrant were recorded by a video camera and analyzed by a tracking software (EthoVision XT 15, Noldus Information Technology, Wageningen, The Netherlands).

### 2.3. Long-Term Potentiation Recording at CA3-CA1 Hippocampal Synapses

**Figure 6:**
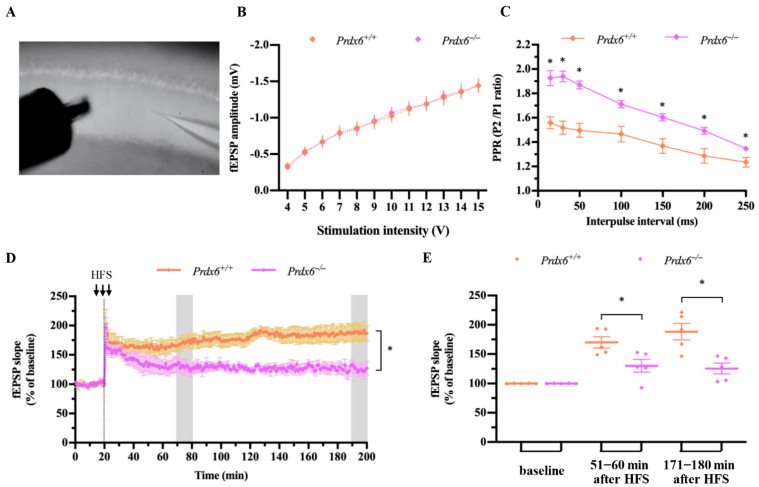
Impaired hippocampal LTP in female *Prdx6^−/−^* mice. (**A**) Representative image for the positions of stimulus and recording electrodes in the hippocampal CA1 region. (**B**) Input–Output curve for fFPSP amplitudes (*n* = 8 slices/ 5 mice in each group). (**C**) the paired–pulse facilitation (PPF) ratio, delivered throughout the seven selected interstimulus intervals (15, 30, 50, 100, 150, 200, and 250 ms) (*n* = 8 slices/ 5 mice in *Prdx6^+/+^* group and *n* = 9 slices/ 5 mice in *Prdx6^−/−^* mice). (**D**) The averaged fEPSP traces of *Prdx6^+/+^* and *Prdx6^−/−^* mice (*n* = 5 mice/group). The gray boxes represent the regions of the last 10 min of the first and third hours. (**E**) The averages of the percentage of normalized fEPSP slopes from the last 10 min of the first and third hours. All data are presented as mean ± SEM., * *p* < 0.05, mixed-design repeated measure ANOVA followed by *Bonferroni*’s post hoc test with unpaired Student’s *t*-test for individual differences between groups within each time point. fEPSP, field excitatory postsynaptic potential; PRDX6, peroxiredoxin 6; PPR, paired-pulse ratio; HFS, high frequency stimulation.

A different batch of mice was used to investigate pre-and postsynaptic function of hippocampal neurons. Mice were anesthetized with isoflurane and decapitated to remove the brain. The brain tissues were immediately washed with ice-cold artificial cerebrospinal fluid (ACSF) (117 mM NaCl, 4.7 mM KCl, 2.5 mM CaCl_2_, 1.2 mM MgCl_2_-6H_2_O, 1.2 NaH_2_PO_4_-H_2_O, 25 mM NaHCO_3_, and 11 mM D-glucose). The brain tissues were horizontally sliced by vibrating microtome (Leica VT1000 s, Leica Biosystem Inc., Nussloch, Germany) with 350 µm thickness in oxygenated (95% O_2_/ 5% CO_2_) ACSF. Hippocampal slices were incubated in ACSF at 30 °C for 2–3 h before recording. Then the hippocampal slices were transferred to a recording chamber for extracellular LTP recording. The recording procedure was conducted as described in our previous publication [8] . Slices were perfused with ACSF at a speed of 20 rpm. The glass pipettes were pulled on a vertical micropipette puller and filled with ACSF. Then, the recording electrode was placed at the dendritic layer of hippocampal CA1 for recording the excitatory postsynaptic field potentials (fEPSPs). The unipolar tungsten electrode was used as a stimulus electrode. The locations of recording and stimulating electrodes are demonstrated in Figure 6A. The stimulating intensity was adjusted between 4–15 V for each slice to obtain input/output (I/O) response. Paired–pulse facilitation (PPF) was recorded three times at interpulse intervals (IPIs) of 15, 30, 50, 100, 150, 200, and 250 milliseconds, and an average for three recordings at each IPI was presented. The fEPSPs were elicited to approximately 45–50% of the maximal response. Baseline fEPSPs were evoked every 20 s for 20 min, followed by high-frequency stimulation (HFS), which delivered three trains of 100 pulses at 100 Hz for 60 s. Then, fEPSPs were stimulated every 20 s for an additional 180 min to measure the early and late phases of hippocampal long-term potentiation (LTP). The downward slope of fEPSPs was measured using Axon pCLAMP 11 electrophysiology data acquisition and analysis software. Recordings were amplified using an Axon Multiclamp 700B amplifier (Axon Instruments, Foster City, CA, USA), acquired at 10 kHz by an Axon Digidata 1550 B plus HumSilencer (Axon Instruments, Foster City, CA, USA). All signals were filtered at 1 kHz.

### 2.4. Statistics Analysis

All data are presented as mean ± the standard error of the mean (SEM), with statistical significance at *p*-value < 0.05 by analysis with SPSS (version 25, IBM Corporation, New York, NY, USA). All graphs were plotted using GraphPad Prism version 8. The Shapiro–Wilk test was used to assess the data normality. Student’s *t*-tests were applied to compare two independent data groups with normal distribution. In contrast, data without normal distribution was examined with Mann–Whitney U test. The differences in the learning ability of TFC, hidden platform trials, and time spent in each quadrant during the probe trial were assessed with mixed-designed repeated measure ANOVA and analyzed by *Bonferroni*-corrected *t*-test. Sample sizes are described in figure legends.

## 3. Results

### 3.1. Locomotor Functions of Female Prdx6^−/−^ Mice Are Similar to Those of Prdx6^+/+^ Mice

The open field test was used to measure the locomotion function based on their spontaneous exploratory behavior in an open field maze (Figure 2A) [19,20]. The female *Prdx6^−/−^* mice showed no significant difference in the total distance traveled (Figure 2B) [*U* = 25, *p* = 0.171] and moving speed (Figure 2C) [*t*_16_ = 1.614, *p* = 0.126] compared with *Prdx6^+/+^* mice after 10 min of exploration.

We further assessed their motor coordination by rotarod test (Figure 2D). This test is dependent on the functions of the basal ganglia and cerebellum [21,22]. *Prdx6^−/−^* mice were able to remain on the rotating rod like their wild-type littermates. No significant difference was recorded in the latency to fall (Figure 2E) [*t*_16_ = −1.523, *p* = 1.147] and speed at falling time point (Figure 2F) [*t*_16_ = −1.523, *p* = 1.147] between female *Prdx6^−/−^* and *Prdx6^+/+^* mice. These results indicated that the lack of PRDX6 did not affect motor functions in female mice.

### 3.2. Female Prdx6^−/−^ Mice Exhibited a Higher Degree of Anxiety-Like Behavior

A decrease in antioxidant enzymes has been implicated in abnormal emotional responses [23]. We also evaluated the emotional responses, including anxiety and fear, of female mice lacking the *Prdx6* gene. After a 10 min test period in an open field maze (Figure 2A), *Prdx6^−/−^* mice showed a significant decrease in the number of times entering into the center zone (Figure 3A) [*U* = 12.5, *p* = 0.013] and in time spent in the center (Figure 3B) [*t*_16_ = 3.389, *p* = 0.004] and middle (Figure 3C) [*U* =18, *p* = 0.047] zones compared with *Prdx6^+/+^* mice. It is worth noting that the percentage of time spent in the outer area of the open field arena was significantly higher in female *Prdx6^−/−^* mice (Figure 3D) [*U* = 18, *p* = 0.047] compared with their wild-type littermates, suggesting higher anxiogenic behavior.

To confirm the increased anxiety-like behavior of the female *Prdx6*^−/−^ mice, the mice were trained in a different anxiety test, the light-dark transfer test (Figure 3E). We analyzed the ambulatory ability of the mice in the light compartment. We found that *Prdx6^−/−^* mice showed similar traveling distance (Figure 3F) [*t*_16_ = −0.387, *p* = 0.704] and moving speed (Figure 3G) [*t*_16_ = 1.004, *p* = 0.33] to those of their wild-type littermates. We found a significant decrease in the percentage of risk assessment behavior of female *Prdx6^−/−^* mice (Figure 3H) [*t*_16_ = 3.335, *p* = 0.004] compared with the *Prdx6^+/+^* controls. No significant difference was found between groups in the number of entries to the light compartment (Figure 3I) [*t*_16_ = 0.215, *p* = 0.832] and time spent in the light compartment (Figure 3J) [*t*_16_ = −1.668, *p* = 0.115]. These results indicate that female *Prdx6^−/−^* mice displayed higher levels of anxiety-like behavior than their wild-type littermates.

### 3.3. Female Prdx6^−/−^ Mice Exhibited an Excessive Fear Response

Females are prone to experiencing fear-associated disorders [24]. We further investigated the impact of PRDX6 deficiency in female mice on trace fear memory by conducting trace fear conditioning (Figure 4A). The freezing percentages were used to indicate fear learning ability and contextual fear memory. On the training day, mice from both groups learned to associate cues with electric foot shocks. No significant difference was found in their learning ability (Figure 4B) [*F*_(1,16)_ = 1.658, *p* = 0.832] and total freezing percentage during training sessions (Figure 4C) [*t*_16_ = −1.135, *p* = 0.273]. We subjected the mice to the same conditioning chamber to test their contextual fear memory. We found that female *Prdx6*^−/−^ mice exhibited a significantly increased freezing behavior (Figure 4D) [*t*_16_ = −2.663, *p* = 0.017] compared with female *Prdx6^+/+^* mice. It indicated that lack of the *Prdx6* gene promotes excessive fear memory in female mice.

### 3.4. Female Prdx6^−/−^ Mice Exhibited Lowered Spatial Memory Retention in Morris Water Maze (MWM) Test

We further investigated the spatial memory performance of female *Prdx6^−/−^* mice using the MWM test (Figure 5A). No statistical difference was recorded for escape latency (Figure 5B) [*t*_30_ = −0.802, *p* = 0.429] and swimming speed (Figure 5C) [*t*_30_ = −1.041, *p* = 0.306] in the visible platform trial between the two genotypes, suggesting normal visual ability and sensorimotor function of the female *Prdx6*^−/−^ mice. Both groups successfully learned the task (Figure 5D) [*F*_(2.74, 82.27)_ = 53.54, *p* = 0.000]. Female *Prdx6^−/−^* mice and their wild-type littermates performed similarly on spatial learning tasks, as indicated by similar escape latencies to reach the hidden platform during training days 1 to 5 (Figure 5D) [F_(1, 30)_ = 1.576, *p* = 0.219]. In the probe test, mice were allowed to swim in the maze for 60 s. We found that both female *Prdx6^−/−^* and *Prdx6^+/+^* mice spent longer time in the target quadrant than the other quadrants (Figure 5E) [*F*_(2.110, 33.758)_ = 12.943, *p* = 0.000; F_(2.129, 29.812)_ = 33.588, *p* = 0.000, respectively]. However, female *Prdx6*^−/−^ mice spent less time in the target quadrant than *Prdx6^+/+^* mice did (Figure 5E) [*t*_30_ = 2.499, *p* = 0.018], and their swimming traces were not as confined in the target quadrant as those of *Prdx6*^+/+^ mice (Figure 5F), indicating lower spatial memory retention.

### 3.5. Female Prdx6^−/−^ Mice Demonstrated Reduced Hippocampal LTP

Since the female *Prdx6^−/−^* mice demonstrated a lowered spatial memory retention, we next asked whether their hippocampal synaptic function was normal. We used field recording at Schaffer collateral CA1 synapses of hippocampal slices (Figure 6A) to evaluate the synaptic function in female *Prdx6*^−/−^ mice. We first measured the relationship between the amplitude of field potentiation and the stimulus intensity (input-output or I-O curve). The input–output curve indicated that the basal synaptic transmission in the hippocampal CA1 of female *Prdx6^−/−^* mice was similar to that of their *Prdx6^+/+^* littermates. No significant difference was found in the amplitudes of field excitatory postsynaptic potentials (fEPSP) elicited by stimulating intensity ranging from 4 to 15 volts (Figure 6B).

We then assessed short-term, activity-dependent synaptic plasticity [25] by performing paired–pulse facilitation (PPF) on Shaffer collateral inputs onto hippocampal CA1 neurons. PPF was significantly stronger in female *Prdx6*^−/−^ than *Prdx6*^+/+^ mice in response to synaptic inputs (Figure 6C) [15-ms interpulse interval (IPI), *t*_15_ = −4.653, *p* = 0.000; 30-ms IPI, *t*_15_ = −5.894, *p* = 0.000; 50-ms IPI, *t*_15_ = −5.473, *p* = 0.000; 100-ms IPI, *t*_15_ = −3.346, *p* = 0.004; 150-ms IPI, *t*_15_ = −3.390, *p* = 0.004; 200-ms IPI, *t*_15_ = −3.039, *p* = 0.008; 250-ms IPI, *t*_15_ = −2.429, *p* = 0.028].

We next recorded the postsynaptic response of hippocampal CA1 neurons after high-frequency stimulation (HFS). The slope of fEPSPs recorded during baseline, 1st hour, and 3rd hour of LTP were shown in Figure 6D. The average slopes for baseline were not different between the two genotypes (Figure 6D,E) [*t*_8_ = 0.460, *p* = 0.658]. Interestingly, the slopes of fEPSPs were significantly lower in the female *Prdx6*^−/−^ mice than those recorded from their wild-type littermates at 1 h (Figure 6D,E) [*t*_8_ = 2.763, *p* = 0.025] and 3 h (Figure 6D,E) [*t*_8_ = 3.737, *p* = 0.006] after HFS. These results indicated that loss of PRDX6 causes impaired hippocampal synaptic plasticity.

## 4. Discussion

**Figure 7:**
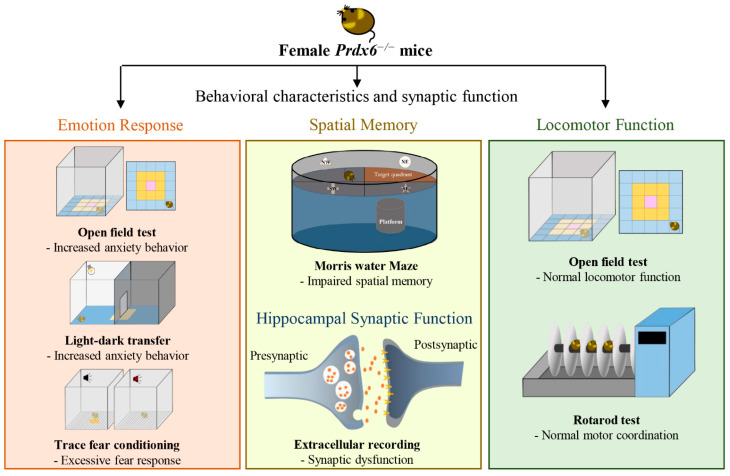
Graphical summary for the behavioral and synaptic phenotypes of female *Prdx6^−/−^* mice. The female *Prdx6^−/−^* mice exhibited high emotional responses, including anxiety-like behavior and enhanced fear expression (orange box). These mutant mice also displayed impaired spatial memory and hippocampal synaptic function (yellow box). Normal locomotion and motor coordination were also observed in female *Prdx6^−/−^* mice (green box).

Figure 7 summarizes the findings of this study. The present study found that female *Prdx6^−/−^* mice exhibited higher anxiety-like behaviors and expressed excessive fear responses (Figure 7, orange box) despite their normal locomotor functions (Figure 7, green box). Additionally, female *Prdx6^−/−^* mice were impaired in spatial memory retention, accompanied by reduced synaptic transmission in the hippocampus (Figure 7, yellow box). The results confirm our previous findings that PRDX6 plays a critical role in emotional response and memory performance [8]. To our best knowledge, this is the first report regarding the influence of gender factors on anxiety levels in mice lacking the *Prdx6* gene.

Like male *Prdx6^−/−^* mice, female *Prdx6^−/−^* mice also demonstrated enhanced contextual fear [16], impaired spatial memory [8], and reduced hippocampal LTP [8]. However, female *Prdx6^−/−^* mice exhibited higher levels of anxiety-like behavior, while male *Prdx6^−/−^* mice were not more anxious than their wild-type littermates [8,16]. Further experiments are required to help understand the factors involved in the gender difference related to the degree of anxiety experienced. It has been reported that various anxiety disorders and neurodegenerative diseases such as Alzheimer’s disease (AD) have a higher incidence in females [26,27]. Alzheimer’s disease is usually related to higher anxiety levels [27] and spatial memory impairment [28]. Several studies have demonstrated that gender factors contribute to phenotypic differences [20,24]. One of the most important factors is the different protein expression levels in the female and male brains [29], which would affect related protein activation or signaling transduction, leading to distinct behaviors. PRDX6 expression levels are different in males and females [10]. Its functions are also distinct under some pathological conditions [14,30]. In the present study, we did not observe changes in locomotor functions. Therefore, the effect of locomotion on the behavioral phenotypes of female *Prdx6^−/−^* mice is excluded. Without giving any stressor, female *Prdx6^−/−^* mice demonstrate a higher level of anxiety-like behavior, while male *Prdx6^−/−^* mice do not show enhanced anxiety behavior [16] unless receiving restraint stress [9]. A recent study published by Gu SM et al. revealed that female *Prdx6* transgenic (Tg) mice that overexpress PRDX6 exhibited less anxiety-like behavior in an open field and elevated plus-maze test [29]. It is worth noting that this phenotype was not found in male *Prdx6* Tg mice, suggesting a different influence level of PRDX6 in anxiety-like behavior between females and males. The authors also found that the 5-hydroxytryptamine receptor 2C (*Htr2c*) mRNA, coding a serotonin receptor, was upregulated in female *Prdx6* Tg mice, and the levels were higher than those in male Tg mice [29]. This G-protein-coupled serotonin receptor, HTR2C, is involved in demonstrating anxiety-like behavior. Mice lacking the *Htr2c* exhibit an anxiogenic phenotype [30]. These findings suggest that loss of PRDX6 may lead to low levels of HTR2C, which subsequently promotes anxiogenic behavior. This may account for the behavioral phenotype of female *Prdx6^−/−^* mice. It is not clear whether the anxiety-like behaviors are due to abnormal development or the dysfunction of other organs since PRDX6 is expressed all over the body [7]. We also observed grossly normal development of the *Prdx6^−/−^* mice [7,16]. Although PRDX6 is involved in the cell cycle and proliferation [31], the morphology of many vital organs, such as the heart, lungs, and liver, remains intact in these knockout mice [7]. Therefore, these changes in behavioral phenotypes may be primarily due to the effect of PRDX6 on brain function. Further studies are necessary to delineate the relationship between PRDX6, serotonin, and HTR2C regarding anxiety behavior.

Anxiety is different from fear response in many aspects, such as the etiologies, duration times, and behavioral patterns. Anxiety is typically a response to an unknown threat, while fear is involved in known external danger [32]. We found that female *Prdx6^−/−^* mice showed a higher anxiety-like behavior than males; however, both genders exhibited increased fear responses [16]. Thus, we could assume that the molecular mechanism underlying this higher anxiety-like behavior may depend on sex-related signaling pathways.

Spatial memory is a form of episodic memory involving the encoding and recalling of spatial cues associated with environments [33]. Impairment of spatial memory is one of the most common symptoms of AD, psychiatric disorders, and aging [34,35,36]. The hippocampus is the brain area that plays a critical role in synaptic regulation and the formation of emotional and spatial memories [37]. PRDX6 expression has been found throughout the brain, including in the hippocampus [38]. And its expression is associated with various neurodegenerative diseases such as Parkinson’s disease [39] and AD [40]. It is known that PRDX6 may exert either neuroprotective or neurotoxic effects due to its functional diversity and its expression throughout brain subregions [14,41]. A proteomic study revealed that downregulation of PRDX6 in the dentate gyrus (DG) of the hippocampus is associated with spatial memory deficits in aging rats [42]. Whether the impaired spatial memory of the *Prdx6^−/−^* mice found in this study and previous publications results from a lack of PRDX6 in the DG subregion requires a further gain-of-function study on *Prdx6^−/−^* mice.

We also found that impaired spatial memory retention is accompanied by reduced synaptic strength in female *Prdx6^−/−^* mice, the same as the male *Prdx6^−/−^* mice [8]. In the present study, we detected that PPF was increased, and hippocampal-LTP was reduced in the female *Prdx6^−/−^* mice. The ratio of PPF represents short-term plasticity associated with successively increased calcium levels in the presynaptic terminal. Increased calcium levels help enhance the probability of transmitter release [25]. PRDX6 can help maintain calcium homeostasis by limiting intracellular ROS [43]. Since the instability of extracellular calcium can lead to changes in the probability of neurotransmitter release and, in turn, cause an increase in PPF, lack of PRDX6 might lead to an imbalance in calcium homeostasis and result in enhanced PPF [44]. In vitro studies have revealed a relation between LTP magnitude and changes in PPF ratio, indicating presynaptic contributions to LTP. Our recent study also found that male *Prdx6^−/−^* mice were impaired in spatial memory, along with reduced LTP [8]. The aiPLA_2_ activity of PRDX6 may regulate several important signaling pathways related to LTP induction in the hippocampus [45]. Moreover, PRDX6-aiPLA2 activity can also induce the NADPH oxidase 2 (NOX2) pathway, which is important for synaptic plasticity [46]. NADPH mutant mice exhibit hippocampal-dependent memory deficits and LTP impairment [47]. These molecules may be related to the LTP reduction and spatial memory impairment of female *Prdx6^−/−^* mice. Further molecular study will help verify molecular interactions regarding PRDX6’s function in synaptic plasticity.

## 5. Conclusions

In conclusion, the present study found that female *Prdx6^−/−^* mice demonstrated enhanced fear responses and lowered spatial memory retention as shown in male *Prdx6^−/−^* mice. Moreover, female *Prdx6^−/−^* mice exhibited higher anxiety-like behaviors, which is not found in male *Prdx6^−/−^* mice. Our findings may help account for gender differences regarding PRDX6’s function in anxiety.

## Data Availability

Data is contained within the article.
